# Malignant renal hemangiopericytoma: a case report

**DOI:** 10.11604/pamj.2022.41.36.31841

**Published:** 2022-01-13

**Authors:** Singgih Winoto, Muhammad Asykar Palinrungi, Khoirul Kholis, Syakri Syahrir, Syarif, Abdul Azis, Muhammad Faruk

**Affiliations:** 1 Department of Surgery, Faculty of Medicine, Hasanuddin, University, Makassar, Indonesia,; 2 Division of Urology, Department of Surgery, Faculty of Medicine, Hasanuddin University, Makassar, Indonesia

**Keywords:** Malignant hemangiopericytoma, kidney, fibrous tumor, radical nephrectomy, case report

## Abstract

Malignant hemangiopericytoma (HPC) is an uncommon disease first described by Stout and Murray in 1942. Patients with suspected renal HPC on admission sometimes complain of low back pain, hematuria, or hypertension. A combination of histochemical and anatomo-pathologic examinations is necessary to confirm the diagnosis of renal HPC. We report the case of a 41-year-old female patient who had persistent painful nodular lesion at the right lower back and gross hematuria. Based on analyses of clinical symptoms and signs plus radiological a laboratory examination, she was diagnosed with renal cell carcinoma of the right kidney. She subsequently underwent open right radical nephrectomy via transperitoneal approach. The patient was discharged from hospital in good condition on Day 5 of care after surgical intervention. Malignant HPC of the kidney is an uncommon disease that can be diagnosed based on multislice computerized tomography angiography plus histopathological examination using the periodic acid shift method. Management of malignant renal HPC requires radical nephrectomy followed by chemotherapy. This case study provides important preliminary data for further studies of patients with renal HPC in Indonesia.

## Introduction

Hemangiopericytoma (HPC), also known as solitary fibrous tumor, is a rare, highly vascularised, soft tissue tumor originated from pericytes, which are mesenchymal cells lining capillary walls [1]. HPC very often occurs in the pelvis, head, and neck, as well as in meninges; it is rarely found in the kidneys [2]. In 1942, Stout and Murray first described HPC in the literature as a complex neoplasm composed of capillaries and perivascular cells but lacking organoids. HPC is a locally damaging tumor with different malignant potential. Haematogenous spread to the lungs, lymph nodes, and bones can occur [2,3].

By 2009, 41 case reports including patients with renal HPC were reported in the literature [4]. The most frequent type of renal HPC was found in capsular tissues or connective tissues of the peripelvic interstitium. Most cases are suspected of having renal cell carcinoma [3]. Patients with suspected renal HPC on admission sometimes complain of low back pain, hematuria, or hypertension. Histochemical and anatomo-pathological examinations are necessary to confirm the diagnosis of renal HPC [5]. To our knowledge, this is the first study of a patient with malignant HPC of the kidney in Indonesia.

## Patient and observation

**Patient information**: a 40-year-old woman was admitted to our institution complaining of persistent painful nodule at the right lower back. The pain occasionally spread to the right upper abdomen and the solar plexus. She experienced signs of terminal gross hematuria for one week before admission to the hospital. The patient had no close relatives with the same clinical manifestations.

**Clinical findings**: upon physical examination, blood pressure was 150/100 mmHg, heart rate was 72 beats/min, and body temperature was 36.8°C.

**Timeline of current episode**: she had a history of hypertension and took 25 mg of captopril once a day. She reported no family history of HPC and was not a smoker and didn´t drink alcohol.

**Figure 1:**
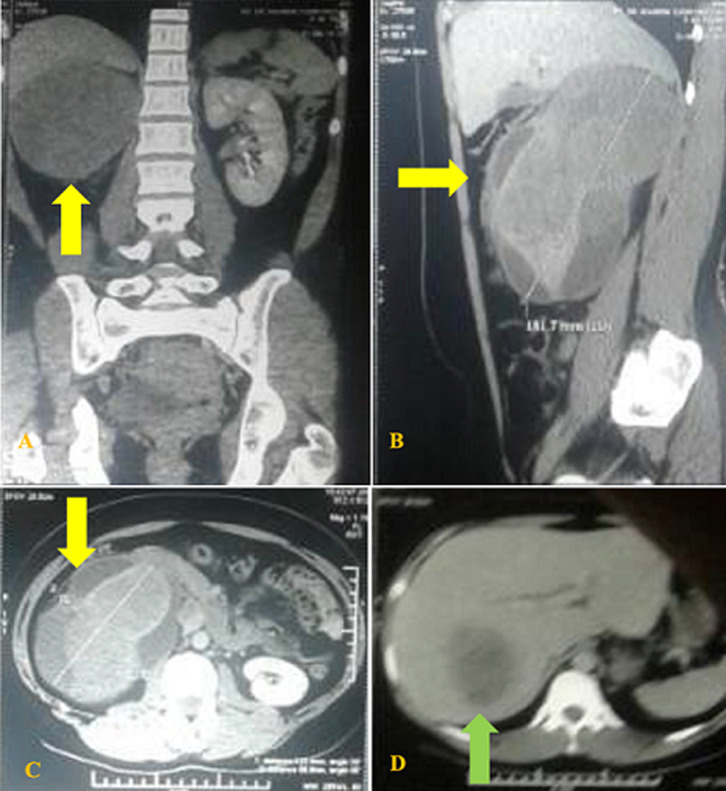
multislice computerized tomography urography and abdominal contrast showing a nodular solid lesion of size 12.3 cm x 8.9 cm x 18.1 cm at the right kidney (yellow arrow) with a suspected metastasis to the liver (green arrow)

**Figure 2:**
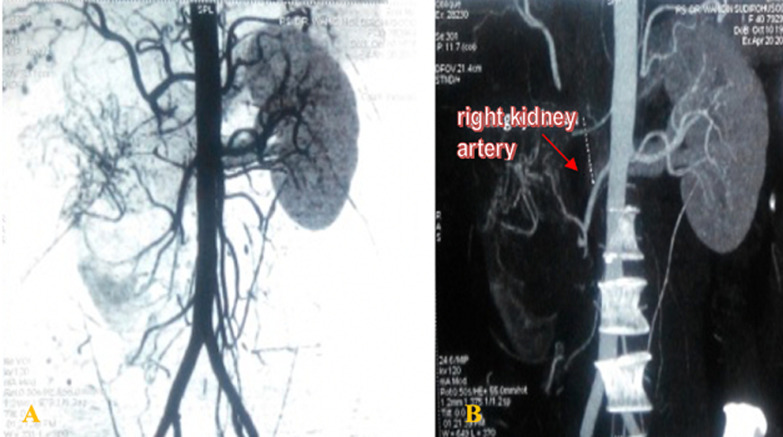
multislice computerized tomography angiography showing the kidney immediately adjacent to the suspected kidney cell carcinoma

**Diagnostic assessment**: laboratory tests showed a serum hemoglobin (hb) level of 11.3 g/dL, leukocytes 11,600 mm^3^, thrombocytes 419,000 mm^3^, erythrocyte sedimentation rate 69 mm/hr, blood-urea concentration 23 mg/dL, and creatinine 0.7 mg/dL; all other blood test results were normal. Urinalysis showed urine pH 6.0, leukocytes were zero, negative protein, and blood 3+/200. Multislice computerized tomography (MSCT) urography with contrast medium injection showed oval- shaped, well-demarcated lesion with a solid mass and a size of 12.3 cm x 8.9 cm x 18.1 cm in the perirenal fat of the right renal fossa (Figure 1). Abdominal examination using MSCT angiography found hypodense mass at the right lobe of the liver and a dense mass at the right kidney. A slight hypodense mass (37 HU; 65 HU post- contrast) was found in the right renal artery with arterial feeding, whereas neither a dense mass nor arterial feeding was found in the left kidney (Figure 2). The patient had no hydronephrosis.

**Diagnosis**: based on the analyses of clinical symptoms and signs, laboratory results, and radiological examinations, the patient was diagnosed with renal cell carcinoma of the right kidney with suspected metastasis to the liver.

**Figure 3:**
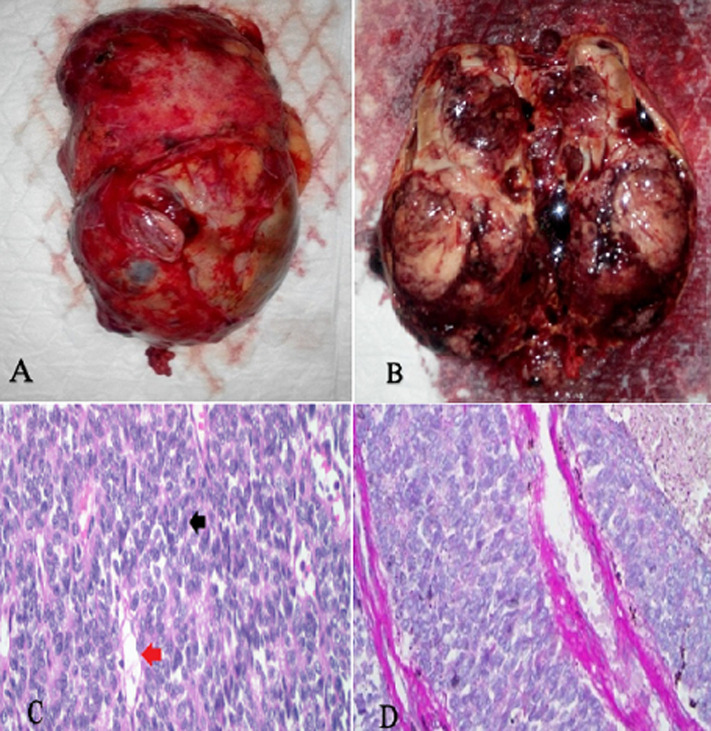
A) kidney tumor with intact capsule; B) tumor sliced into two parts; C) histological features of the surgical specimen with hematoxylin eosin staining, showing a tumor cell (black arrow) and a blood capillary (red arrow); D) periodic acid shift staining depicting blood capillaries of the kidney basal membrane (40x magnification)

**Therapeutic interventions**: consequently, the patient underwent open right radical nephrectomy via transperitoneal approach. The surgical specimen was surrounded by an intact capsule measuring 12 cm x 8 cm x 15 cm; it weighed 500 g and was characterized by a whitish pink color on the external surface (Figure 3A, Figure 3B). Histopathological examinations of hematoxylin and eosin (H&E) stained tissue from the right kidney was performed. In specimen tissues the tubulus and glomerulus appeared to have no specific abnormalities. The tumor nest contained oval nuclei, spindle cells with blunt edges, crude chromatin, and abundant mitoses (> 30/10 hpf). Nuclei surrounded blood capillaries with perivascular hyalinization (Figure 3C). Histochemical staining was subsequently carried out using periodic acid shift (PAS) method; red color (PAS+) showed that the tumor mass was localized in the external wall of blood capillaries (Figure 3D). Based on these findings, we diagnosed the patient with malignant HPC of the kidney.

**Follow-up and outcome of interventions**: the patient was in good condition after surgery, and she was discharged on day 5 of care after surgical intervention.

**Patient perspective**: chemotherapy was then recommended, but the patient refused it; she died one year later due to progression of the disease during a several-month follow-up period.

**Informed consent**: the parents/guardian of the patient provided informed consent for the publication of her clinical data. The presented data are anonymized and risk of identification is minimal.

## Discussion

Malignant renal HPC is an extremely rare soft-tissue vascular tumor caused by the uncontrolled proliferation of pericytes [3]. These cells were first described by Zimmerman in 1923 [2,3,6]. Mean age of patients with renal HPC is 40.3 years at the time of diagnosis; patients with renal HPC are slightly younger than patients with other types of renal cell carcinoma [3,7]. In addition, the incidence of malignant renal HPC is not significantly different between males and females [4]. Thus, the case presented here is not atypical in age or sex. In the case reported here, prominent clinical manifestations associated with advanced malignant renal HPC were non-specific: a painful nodular lesion at the right lower back and hematuria. These data are in line with other studies that have found non-specific symptoms and signs of renal HPC. In 66% of previously reported cases, the earliest common symptom was a painless abdominal tumor [8]. Other reported symptoms included, in ascending order of frequency, hematuria, hypoglycemia, and arterial hypertension [2,4].

In diagnostic imaging, such as ultrasound, MSCT scan, or Magnetic resonance imaging, no special signs distinguish HPC from other renal tumors [3,4]. Nevertheless, renal HPC can be distinguishable, in early arterial phase angiography, by the displacement of the main arteries, the presence of large capillaries surrounding the tumor, and well-demarcated tumor stain [4,8]. Using MSCT angiography, we were able to confirm the diagnosis of renal HPC based on the arrangement disparity and hypervascularization of the main arteries and blood capillaries that surrounded the tumor, as has been demonstrated in other studies. Diagnosis can also be established through a combination of HE histological staining and immunohistochemical staining using the PAS method, with antibodies against CD31, CD34, CD68, and vimentin [9,10]. In the present study, histological examinations using both HE staining and PAS staining showed that the patient had solitary fibrous tumor, which then progressed into malignant renal HPC. The best choice of therapy for patients with malignant renal HPC is surgery following angiographic examination, as surgical removal of early-stage lesion remains the only potential curative therapy available [4,11]. The best treatment option for patients with renal HPC showing no metastasis is radical nephrectomy. The most common site of metastasis is the lungs. In an advanced metastatic tumor (most often in the lungs and liver) along with hematuria, pain, or paraneoplastic syndrome, radical nephrectomy is the only palliative therapy [4,8].

Radiotherapy and chemotherapy are used as adjuvant therapies after surgical intervention. The dose of radiotherapy for renal HPC is 75-90 Gy, although this sometimes results in severe toxicity [8]. The appropriate chemotherapy regimen is a combination of ifosfamide at 1800 mg/m^2^ and etoposide at 100 mg/m^2^ on Days 1-5 of every 3-week cycle, alternately given with a combination of vincristine at 1.4 mg/m^2^, doxorubicine at 75 mg/m^2^, and cyclophospamide at 1200 mg/m^2^ on Day 1 of each 3- week cycle. Following three cycles of chemotherapy, after which the disease should be stable, another six cycles are completed [8,12,13]. In the present study, the patient was scheduled for chemotherapy, but she refused, and her disease was thus not routinely controlled. Several case reports of renal HPC, which is closely related to solitary fibrous tumors, have reported the therapeutic use of interferon, with or without thalidomide [12]. In one patient with malignant solitary fibrous tumor of the kidney and metastatic disease, treatment with interferon achieved stable disease for about 20 months. Some authors have suggested the use of antiangiogenic therapies (bevacizumab, sunitinib, pazopanib, etc.), based on findings of high vascularity and a possible origin from pericytes [14]. One patient with malignant renal HPC who didn´t undergo resection and without metastasis was treated orally with temozolimide at 150 mg/m^2^, on Days 1-7 and Days 15-21 and treated intravenously with bevacizumab at 5 mg/kg on Days 8 and 22, using a cycle of 28 days [12,13]. In the present study, the patient died during the follow-up period, 1 year after she was diagnosed with malignant renal HPC, because the disease progressed after the patient had refused chemotherapy. Long-term survival rates of patients with malignant renal HPC are only moderate and tend to be worse in adults. One study by Enzinger and Smith reported a 10-year survival rate of 70%-77% in cases with 0-3 mitoses/10 hpf; Mac Master *et al*. reported 52% survival among 60 patients; Auguste *et al*. found that 59% and 47% of patients survived 5 and 10 years, respectively; Espan *et al*. reported that 93% and 86% of patients survived 2 and 5 years, respectively [7,11].

## Conclusion

This is the first case of malignant renal HPC reported in Indonesia. Malignant renal HPC is an uncommon disease that can be diagnosed through MSCT angiography plus histopathological examination using PAS method. Management of malignant renal HPC requires radical nephrectomy followed by chemotherapy. The case investigated in this study provides important preliminary data for further studies of patients with renal HPC in Indonesia.

## References

[1] Vasile G, Mancuso C, White R, Hanly AJ, Moore M, Rubenstein R (2020). Rare meningeal-derived malignant hemangiopericytoma/solitary fibrous tumor grade II-III presenting as a subcutaneous mass on the scalp. JAAD Case Reports.

[2] Anakievski D, Kalchev K (2020). Renal hemangiopericytoma in 15 year old female-treated laparoscopically. Urol Case Reports.

[3] Bilici A, Ustaalioglu O, Seker M, Salman T, Igdem A, Celik E (2009). Metastatic renal hemangiopericytoma: a rare case report. Arch Oncol.

[4] Vetorazzo Filho JE, Bahia LAC, Esteves PE, Maron PEG, Vedovato BC, Fernandes R de C (2015). Renal hemangiopericytoma: case report and literature review. Einstein (SÃ£o Paulo).

[5] Riley DS, Barber MS, Kienle GS, Aronson JK, von Schoen-Angerer T, Tugwell P (2017). CARE guidelines for case reports: explanation and elaboration document. J Clin Epidemiol.

[6] Raghani N, Raghani M, Rao S, Rao S (2018). Hemangiopericytoma/Solitary fibrous tumor of the buccal mucosa. Ann Maxillofac Surg.

[7] Brescia A, Pinto F, Gardi M, Maria Vecchio F, Bassi PF (2008). Renal Hemangiopericytoma: Case Report and Review of the Literature. Urology.

[8] Argyropoulos A, Liakatas I, Lykourinas M (2005). Renal haemangiopericytoma: the characteristics of a rare tumour. BJU Int.

[9] Zhao P, Zhu T, Tang Q, Liu H, Zhu J, Zhang W (2015). Immunohistochemical and genetic markers to distinguish hemangiopericytoma and meningioma. Int J Clin Exp Med.

[10] Han Y, Zhang Q, Yu X, Han X, Wang H, Xu Y (2015). Immunohistochemical detection of STAT6, CD34, CD99 and BCL-2 for diagnosing solitary fibrous tumors/hemangiopericytomas. Int J Clin Exp Pathol.

[11] Penel N, Amela EY, Decanter G, Robin Y-M, Marec-Berard P (2012). Solitary Fibrous Tumors and So-Called Hemangiopericytoma. Sarcoma.

[12] Cuello J, Brugés R (2013). Malignant solitary fibrous tumor of the Kidney: report of the first case managed with interferon. Case Rep Oncol Med.

[13] Ljungberg B, Albiges L, Abu-Ghanem Y, Bensalah K, Dabestani S, Fernández-Pello S (2019). European association of urology guidelines on renal cell carcinoma: the 2019 Update. Eur Urol.

[14] Park S Bin, Park YS, Kim JK, Kim MH, Oh YT, Kim KA (2011). Solitary Fibrous Tumor of the Genitourinary Tract. Am J Roentgenol.

